# Durable Antipilling Modification of Cotton Fabric with Chloropyrimidine Compounds

**DOI:** 10.3390/polym11101697

**Published:** 2019-10-16

**Authors:** Xue Dong, Tieling Xing, Guoqiang Chen

**Affiliations:** National Engineering Laboratory for Modern Silk, College of Textile and Clothing Engineering, Soochow University, Suzhou 215123, China; dongxue@suda.edu.cn (X.D.); xingtieling@suda.edu.cn (T.X.)

**Keywords:** cotton fabric, chloropyrimidine compounds, antipilling, surface, modification

## Abstract

Cotton fabric, a natural cellulose material, is widely used in the textile industry for its excellent properties. However, its application in some fields are seriously restricted because of its poor antipilling behavior. In this study, cotton fabrics were modified with 2,4,6-trichloropyrimidine (TLP), 2,4-dichloro-5-methoxypyrimidine (DMP), and 2-amino-4,6-dichloropyridine (ADP). The surface morphology and chemical structure of the modified cotton fabric were characterized by scanning electron microscopy (SEM), Fourier transform infrared spectroscopy (FTIR), and X-ray diffraction (XRD). Furthermore, the antipilling behavior, dyeing properties, thermal properties, and mechanical properties of modified cotton fabric were evaluated. The results showed that chloropyrimidine compounds were successfully grafted onto the surface of the cotton fabric, leading to excellent and durable antipilling activity of grade 3–4 even after 10 washes. Moreover, compared with control cotton fabric, the heat release rate (HRR) and total heat release (THR) of TLP-modified cotton fabric decreased to 173.2 W/g (42.3% reduction) and 11.3 KJ/g (13.7% reduction), respectively. In addition, the increased K/S value of modified cotton fabrics dyed with reactive dyes indicated that the modification can enhance the dyability of cotton fabric. This technique provides a simple and versatile method for improving the antipilling behavior of cellulosic materials and supports further preparation of functional textiles.

## 1. Introduction

Cotton fabric, a green natural cellulose product, is highly popular in our daily life due to its outstanding properties, such as softness, high hygroscopicity, biodegradability, air permeability, and comfort [1,2,3,4,5]. Many interesting and advanced applications related to natural cellulose materials have been reported [6,7,8,9,10]. However, cotton fibers easily unravel and the loose fiber ends form balls on the surface of fabric during the process of dyeing and finishing, wearing, and washing [11], which affects the handle, appearance, and wearability of fabrics. With the improvement of living standards, people have higher and more complete requirements of textile quality and functionality. The poor antipilling behavior of cotton fabric restricts its application in the textile industry. Therefore, it is meaningful to develop an efficient and industrialized method to produce high quality cotton products with excellent antipilling behavior.

Recently, researchers have studied a variety of strategies to improve antipilling behavior, such as enzyme finishing [12], low temperature plasma treatment [13], resin (polyurethane) crosslinking finishing [14], oxidant finishing, inorganic alkali corrosion, silk protein coating [15], sol-gel finishing [16]. These strategies play an important role in reducing the pill formation by stabilizing the protruded fibers inside the yarn and removing the surface nap [17]. Among these strategies, resin crosslinking finishing has become a popular method due to its remarkable properties, low cost, and high efficiency [18]. Acrylate copolymer, polyurethane, and polyacrylamide are commonly used as resin crosslinking agents for textiles [19,20,21]. However, significant changes in the mechanical properties and moisture permeability of acrylate copolymer-coated fabrics limit their application. Fabrics coated with modified polyacrylate showed good softness but poor wear resistance [22,23]. Moreover, the stiffness of waterborne polyurethane modified fabric was increased, indicating that the wearing comfort decreased [24]. In addition, common resin finishing methods usually require high temperatures curing process to produce chemical crosslink reactions, which leads to yellowing appearance, strength reduction, and poor handle of fabrics [25].

Chloropyrimidine compounds are heterocyclic compounds containing pyrimidine rings that have been successfully applied in various fields due to their strong biological and pharmacological activities [26]. In particular, halogenated pyrimidine-reactive dyes are widely used in the dyeing industry. However, as far as we know, little work has been done on their application in textile modification.

In the present study, we explored a highly stable emulsion of chloropyrimidine compounds to improve the antipilling behavior of cotton fabric without affecting its wearability. The optimal modification process conditions were investigated to obtain durable pilling resistance of cotton fabrics. The structure and surface morphology of modified cotton fabrics were characterized, the antipilling behavior, mechanical properties, thermal properties, and dyeing properties were also tested. Furthermore, the washing durability test was conducted to evaluate the chemical crosslinking fastness between chloropyrimidine compounds and cotton fabrics.

## 2. Materials and Methods

### 2.1. Materials and Reagents

Plain woven and bleached 100% cotton fabric (106 g/m^2^) was purchased from Suzhou Yunzhi Textile Co., Ltd. (Suzhou, China). The cotton fabrics were used after soap boiling at 100 °C for 60 min. Chloropyrimidine compounds 2,4,6-trichloropyrimidine, 2,4-dichloro-5-methoxypyrimidine, and 2-amino-4,6-dichloropyridine were supplied by Shanghai Adama Reagent Co., Ltd. (Shanghai, China). Sorbitan monolaurate (Span 20), polyoxyethylene dehydrates sorbitol monooleate (Tween 80), sodium chloride (NaCl), and sodium carbonate (Na_2_CO_3_) were supplied by Shanghai Aladdin Biochemical Technology Co., Ltd. (Shanghai, China). Foam-free powder was purchased from Shenggong Biological Engineering (Shanghai) Co., Ltd. The reactive dyes (Red 3BF, Yellow 3RF, and Blue KN-R) was provided by Shanghai Anoky Group Co., Ltd. (Shanghai, China). All the reagents were analytically pure and used without any further purification.

### 2.2. Preparation and Characterization of Dispersed Emulsions

**Figure 1:**
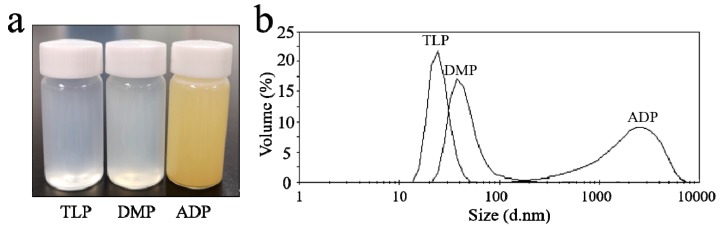
(**a**) Photograph of TLP, DMP, and ADP dispersed emulsions at a concentration of 3 g/L (72 h); (**b**) Droplet size distribution of TLP, DMP, and ADP dispersed emulsions.

Stable and durable dispersed emulsions of chloropyrimidine compounds were prepared by the high shear emulsification method. Briefly, span 20 (20% o.w.t) and tween 80 (30% o.w.t) were added into 100 mL water and emulsified for 30 min at a speed of 10,000 rpm. During the process, 9% (o.w.f) chloropyrimidine compounds (TLP, DMP, and ADP) were slowly added into the solution. Afterwards, the emulsions were diluted by adding water so that a 3 g/L final solution was obtained. Finally, foam-free powder, catalyst, and alkali agent was added into each diluent and stirred evenly to prepare the homogeneous chlorpyrimidine compound dispersion. The obtained dispersed emulsions were stable for a long time without phase separation. Figure 1a shows the photograph of TLP, DMP, and ADP emulsion at a concentration of 3 g/L.

The droplet distribution in each dispersion at a concentration of 3 g/L was analyzed by Dynamic Light Scattering (DLS) and the results are shown in Figure 1b. The TLP dispersion has the smallest average droplet size of 30 nm compared with the DMP and ADP dispersions, and the average droplet size of the DMP dispersion is about 60 nm, indicating that both TLP and DMP diluted dispersions are polymericnano-suspensions. Although the average droplet size of the ADP dispersion increases to 1.4 μm, the dispersed emulsion remains stable and can meet the requirements of modification process.

### 2.3. Preparation of Chloropyrimidine-Modified Cotton Fabrics

**Figure 2:**
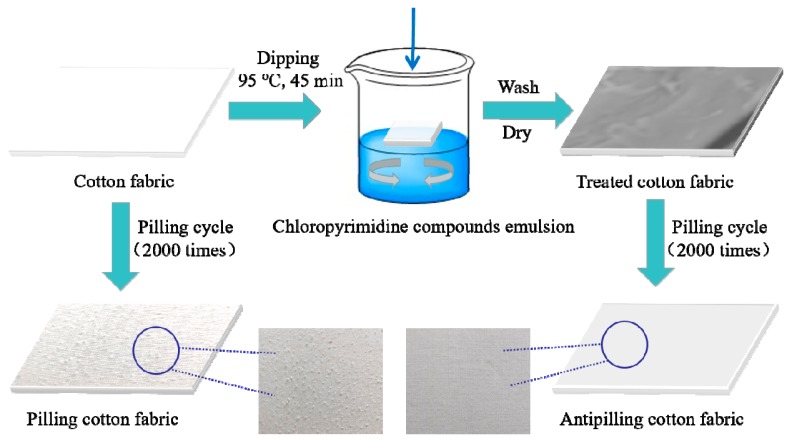
Schematic of the preparation process of the antipilling cotton fabric.

Cotton fabrics were washed and dried before use to ensure that the fabrics were free from contamination. According to the process of Figure 2, the fabric was dipped into dispersion emulsion at different concentrations in an oscillating dyeing machine. Various alkaline agents, different concentration of alkaline agents and sodium sulfate, and different modification time and temperature were designed to explore the optimum application method. The liquor ratio of the fabric and the dispersion emulsion of chloropyrimidine compounds was set to 1:30. The dispersion emulsion was heated from 25 °C to 95 °C at a heating rate of 1–2 °C/min, the fabric was immersed into the emulsion and kept in circulation at 95 °C for 45 min. Finally, the modified cotton fabric was subsequently washed with deionized water at 60 °C for 15 min and then thoroughly rinsed with cold water. Before characterization of the treated fabrics, the samples were dried and stored at room temperature.

### 2.4. Dyeing of Cotton Fabrics

The control and modified cotton fabrics were dyed with reactive dyestuffs by an exhaustion method to observe the dyeing characteristics. The concentration of the dyeing solution was 2% o.w.f, and the ratio of dye solution to cotton weight was maintained at 30:1. A temperature rise was carried out from room temperature to 60 °C at a heating rate of 1–2 °C/min, and the fabric was dyed at 60 °C for 45 min. Sodium carbonate (20 g/L) and sodium chloride (40 g/L) were added gradually in four instalments separated by ten minutes in the dyeing process. After dyeing, the dyed fabrics were withdrawn and washed with warm and cold water until no color change occurred.

### 2.5. Characterization and Measurements

#### 2.5.1. Pilling Characterization

Antipilling behavior of modified and control cotton fabrics was evaluated by modified Martindale method ISO 12945-2: 2000 and PillGrade 3D Fabric Scan & Grade System (SDL Atlas Ltd., MI, USA). The samples were placed under standard laboratory conditions (20 °C and 65% R. H) for 24 h before testing [27]. Two thousand pilling cycles were performed for each sample, and the results of five samples were averaged to obtain the pilling grade.

#### 2.5.2. Surface Morphology and Structure Analysis

The surface morphology of the cotton fibers was observed by desktop scanning electron microscope (Hitachi TM 3030, Tokyo, Japan) and the test voltage was maintained at 15 kV. All samples were conductively plated with gold sputtering before observation. The FTIR spectra of cotton fabrics was recorded with a FTIR instrument (Nicolet 5700, Thermo Fisher Scientific Inc., New York, NY, USA) using KBr pellets in the wave numbers range of 500 cm^−1^ to 4000 cm^−1^, and 16 scans in total. The XRD powder patterns were acquired by using a Philips X’pert-pro MRD (PANalytical Company, Arnhem, The Netherlands) diffractometer with Ni-filtered Cu-Kλ radiation (λ = 0.1542 nm). The 2θ angle of the diffractometer was stepped from 5 ° to 90 ° at a scan rate of 5 °/min.

#### 2.5.3. Thermal Properties

The thermal stability of cotton samples was measured by the Pyris Diamond TG-DTA thermal analyzer (PerkinElmer, Waltham, MA, USA) under an air atmosphere with gas flow rate of 20 mL/min and heating temperature range from 40 °C to 700 °C at a heating rate of 10 °C/min [28]. The heat release property of the cotton samples was measured by a FTT0001 Micro Calorimeter Combustion (MCC) instrument (FTT, West Sussex, UK) heated from 75 °C to 750 °C in a nitrogen stream flowing at a linear heating rate of 1 °C. Each sample (about 5 mg) was equipped with a 40 μL alumina pan.

#### 2.5.4. Durability to Washing

The washing durability test for the cotton samples was conducted according to the standard AATCC61-2006 method in the Wash Tec-P Fastness Tester (Roaches International, Kent, UK). Each sample was immersed into a 40 °C solution containing 2 g/L commercial detergent for 10 min, and the liquor ratio was 50:1. After one washing, the fabric was removed, gently squeezed, and rinsed with fresh deionized water [29]. For each new wash cycle, fresh detergents were added to ensure the presence of surfactants in each cycle.

#### 2.5.5. Mechanical Properties

Tensile properties were measured with an Instron 3365 Universal Testing Machine (Instron, MI, USA). The effective gauge length of the cotton sample was 200 mm, and the extension speed was 100 mm/min. The results were the average of five measurements. Bending and surface friction properties were measured by the Kawabata Evaluation System for Fabric (Kato Tech Co., Ltd., Kyoto, Japan). The sample size was 200 mm × 200 mm. The data were collected and averaged statistically with a confidence level of 95%. All samples were conditioned at 20 ± 1 °C and relative humidity 65 ± 5% for 24 h, prior to any further treatment.

#### 2.5.6. Color Measurements and Colorfastness

Color characteristics values (L*, a*, b*) and color depth (K/S) value of the dyed fabrics were investigated by a Hunter Lab Ultra Scan PRO reflectance spectrophotometer with small area view and D65 primary source [2]. The colorfastness of rubbing and washing with soap were determined according to ISO 105-X16 and ISO 105-C10. The samples were washed in standard soap solution at 40 °C for 30 min for washing colorfastness test. The washing and rubbing fastness levels were classified as fading and staining grade ranging from 1 to 5.

## 3. Results and Discussion

### 3.1. Effects of Reaction Conditions on Antipilling Grade of TLP-Modified Cotton Fabric

**Figure 3:**
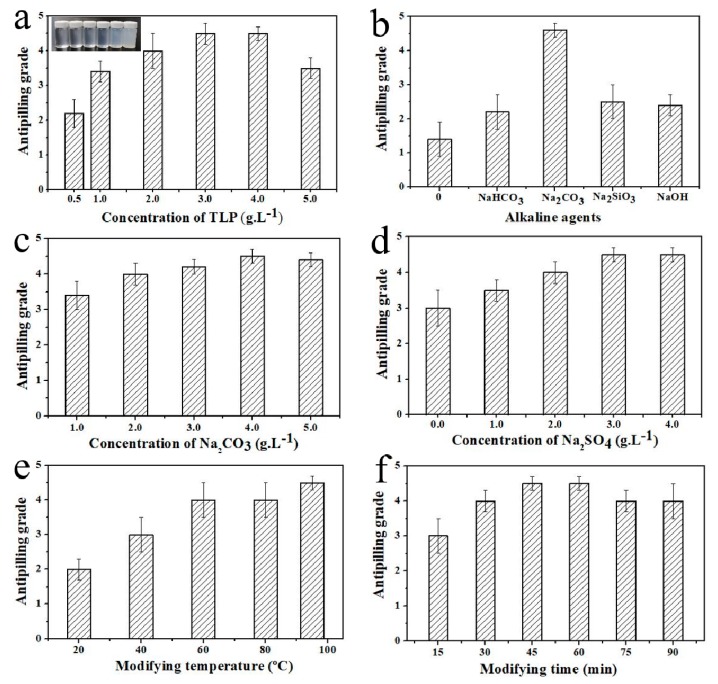
Effect factors in the TLP modification process on antipilling grade of TLP-modified cotton fabric. (**a**) concentration of TLP dispersions; (**b**) kinds of alkaline agents; (**c**) concentration of Na_2_CO_3_; (**d**) concentration of Na_2_SO_4_; (**e**) modification temperature; (**f**) modification time.

In order to prepare cotton fabric with better antipilling behavior, the concentration of TLP dispersions, various alkaline agents, concentration of alkaline agents, concentration of sodium sulfate, and modification time and temperature were considered to optimize the modification conditions of cotton fabric. Figure 3 shows the antipilling grade of each cotton fabric modified with TLP under different reaction conditions. As shown in Figure 3a, the dispersed emulsions change from clear and transparent to translucent and milky suspensions as the concentration of TLP dispersions vary from 0.5 g/L to 5.0 g/L, and they were durable and stable without phase separation. In addition, the antipilling grade of the modified cotton fabric increased gradually with the concentration of TLP dispersions ranging from 0.5 g/L to 3.0 g/L. However, when it reached 4.0 g/L, the antipilling grade remained the same and even decreased a little. Considering resource and cost savings, a TLP concentration of 3.0 g/L was appropriate. The alkaline agent is one of the most important factors in the modification process. As a kind of cellulose fiber, cotton fiber has poor acid resistance but good alkali resistance. It can ionize cellulose anions at pH 8–11. From Figure 3b, it can be clearly seen that Na_2_CO_3_ is the optimum factor, and stronger alkali conditions were not conducive to the covalent crosslinking of cotton fabric with chloropyrimidine compounds. The antipilling grade of the modified cotton fabric did not change markedly when the concentration of Na_2_CO_3_ and Na_2_SO_4_ varied from 2 to 4 g/L (Figure 3c,d); therefore, a concentration of 3 g/L was the most suitable process parameter. It should be pointed out that TLP should have appropriate modification time and temperature when crosslinking with cotton fabric. As shown in Figure 3e and 3f, the temperature of 95 °C and the time of 45 min are the optimum conditions, because longer time or higher temperature can seriously reduce the mechanical properties of cotton fabric.

### 3.2. Surface Morphology and Chemical Structure

**Figure 4:**
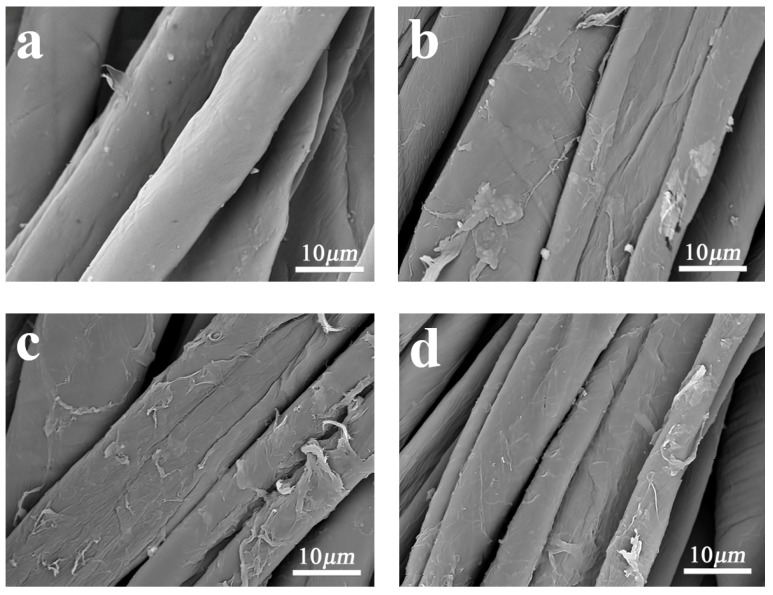
SEM images of (**a**) control cotton fabric; (**b**) TLP-modified cotton fabric; (**c**) DMP-modified cotton fabric; (**d**) ADP-modified cotton fabric. The embedded SEM image in the upper right corner of each photo was the surface morphology of corresponding fabric at a higher magnification.

Surface morphology is a crucial characterization reflecting the pilling property of cotton fabrics. Therefore, the surface morphologies of control and modified cotton fabrics were analyzed by SEM. The SEM images of samples are shown in Figure 4. From Figure 4a, it can be clearly observed that control cotton fibers present typical convoluted and wrinkle-like structure due to the longitudinal fibril structure [30,31]. As shown in Figure 4b–d, a dense layer of film is formed on the surface of the modified fibers and covered the fibers completely, making the fuzz firmly adhered onto the surface of the fibers. Moreover, the cotton fabric surface became rough. Furthermore, compared with control cotton fabric, fibers in modified cotton fabrics held together more tightly and did not easily fall off and separate, because of the crosslinking between fibers and chloropyrimidine compounds and a dense layer of film blocked the interstices between fibers. Therefore, the modified cotton fabrics did not easily fuzz and pill.

**Figure 5:**
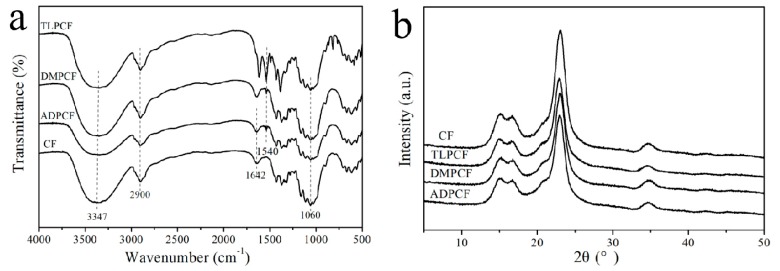
(**a**) FTIR spectra of control cotton fabric and TLP-, DMP-, and ADP-modified cotton fabrics; (**b**) XRD of control cotton fabric and TLP-, DMP-, and ADP-modified cotton fabrics.

The chemical structure of the modified cotton fabrics was analyzed by FTIR and XRD. Figure 5a shows the FTIR spectra of control cotton fabric (CF) and three kinds of modified cotton fabrics (TLPCF, DMPCF, and ADPCF). The characteristic absorption peaks of cellulose backbone at 3347, 2900, 1160, and 1030 cm^−1^ were assigned to the stretching vibrations of O–H, C–H, C–C, and C–O bonds, respectively, in all samples [32,33,34], which indicated that the cellulose chains of cotton did not change after modification by chloropyrimidine compounds. However, a new absorption band of modified cotton fabrics visibly appeared at 1580–1520 cm^−1^ assigned to the stretching vibration of C=C and C=N double bonds of the six-membered ring on chloropyrimidine compounds. Compared with control cotton fabric, a new characteristic peak appeared at about 1540 cm^−1^ attributed to C–N stretching in the TLPCF, DMPCF, and ADPCF spectra [35]. This indicates that a nucleophilic substitution reaction occurs between C–Cl bonds in the pyrimidine ring and the hydroxyl groups of the chain of cellulose macromolecules. Meanwhile, the peak at 3347 cm^−1^ for O–H deformation stretching decreased slightly after the modification with chloropyrimidine compounds, which further confirmed that the nucleophilic substitution reaction existed between cotton fabric and chloropyrimidine compounds.

X-ray diffraction (XRD) was used to study the crystallinity indexes of the control and chemically modified cotton fabrics (Figure 5b). The crystalline zone of cellulose fiber is mainly formed by the ordered arrangement of molecules derived from intermolecular hydrogen bonds. Natural cellulose crystal lattice is called cellulose I [36], and the 2θ angles corresponding to the characteristic peaks of XRD are about 14.8°, 16.4°, 22.6°, and 34.5°, respectively. The results showed that the three kinds of modified cotton samples retained the original crystalline form of pristine cotton fabric regardless of the heterogeneous modification. It was emphasized that the modification occurred only on the active surface or in the amorphous region of cotton fibers. Therefore, the modification of cotton fabrics by chloropyrimidine compounds mainly occurred on the active surface of cotton fabrics.

### 3.3. Antipilling Performance and Washing Durability

The pilling process is mainly related to fiber properties and yarn and fabric structure. The average pill size, pill mass, pill frequency, and volume distribution are also important parameters affecting the quality of cotton fabric [17]. In order to evaluate the antipilling grade accurately, all samples were underwent 2000 pilling cycles. The results presented in Table 1 show that the antipilling grade of the control cotton fabric is 2–3, while the TLP and ADP-modified cotton fabrics are classified as grade 5, indicating that there is almost no pilling on the surface of the modified cotton fabrics.

**Figure 6:**
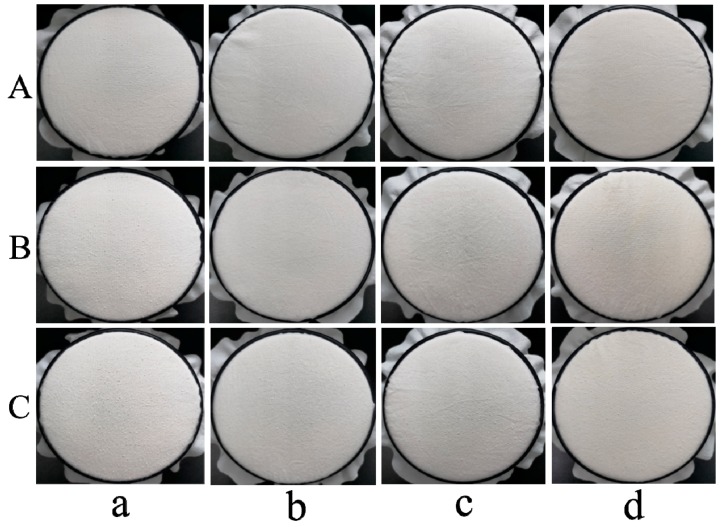
Surface morphology of (**a**) control cotton fabric; (**b**) TLP-modified cotton fabric; (**c**) DMP-modified cotton fabric; (**d**) ADP-modified cotton fabric (**A**. before washing; **B**. after 5 washing cycles; **C**. after 10 washing cycles).

Table 1 and Figure 6 show the pilling grades and the corresponding pictures of the surface morphology of control and modified cotton fabrics before and after five and ten washing cycles, respectively. It can be seen that washing causes pilling, and pilling becomes more serious as the number of washing cycles increases. The pilling grade of control cotton fabric reduced to 1–2 after 10 washing cycles because of the entanglement of loose fibers protruding from the fabric surface. For the cotton fabrics treated with chloropyrimidine compound emulsions, the pilling grade increased to 5 or 4–5 before washing, indicating the good emulsion stability and presence of chloropyrimidine compounds as an effective crosslinking agent. After 10 washing cycles, the antipilling grade decreased by about one and a half grades, but was still higher than the control cotton fabric by two grades. Accordingly, it can be concluded that the cotton fabrics treated with TLP, DMP, and ADP emulsions exhibited excellent antipilling performance and good washing durability compared with control cotton fabric.

### 3.4. Thermal Properties

**Figure 7:**
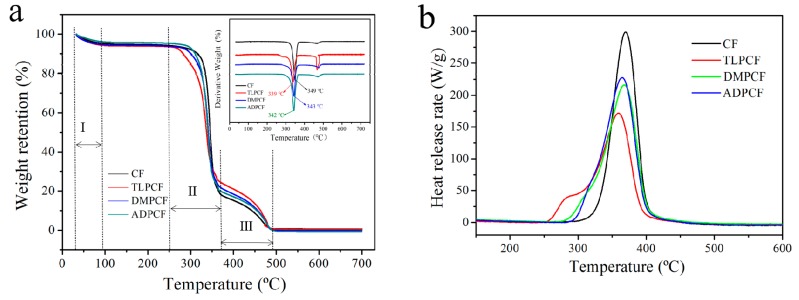
(**a**) TG, DTG, and (**b**) MCC of control and modified cotton fabrics.

The thermal stability of control and modified cotton fabrics was investigated by TGA. As Figure 7a shows, three different stages of mass loss were found in the TG curves. The first stage was observed at below 100 °C, and the mass loss of the fabrics could be attributed to the evaporation of moisture adsorbed in the fabrics [37]. The second stage was observed between 250 °C and 370 °C, and the fabric weight loss was the greatest, corresponding to the decomposition of the cotton fabric into small molecules [38,39]. The stage above 370 °C witnessed the further carbonization of pyrolytic micromolecules caused by the degradation of cotton fabrics. Control cotton fabric displayed the onset of degradation (10% mass loss) at 315 °C and reached 2% char yield of the mass at 700 °C. The decomposition of TLP-, DMP-, and ADP-modified cotton fabric started a little earlier at 282 °C, 300 °C, and 310 °C, respectively, and provided char yields of 1.8%, 1.2% and 0.6%, respectively. From Figure 7a (DTG), control cotton fabric had the fastest decomposition rate at 349 °C and TLP-, DMP-, and ADP-modified cotton had maximum degradation at 339 °C, 343 °C, and 342 °C, respectively. It can be seen that the maximum decomposition rate of TLP-, DMP-, and ADP-modified cotton fabrics was lower than that of control cotton fabric. The results illustrated that the modification might be able to advance the second decomposition stage of the modified cotton fabrics.

Figure 7b shows the heat release curves versus temperature. It can be seen that the cotton fabric treated with the 2,4,6-Trichloropyrimidine (TLP) emulsion system exhibited the best thermal stability, and the heat release rate (HRR) and total heat release (THR) were the lowest compared with the control cotton fabric and the fabrics modified with DMP and ADP. The HRR of TLPCF had a sudden and drastic heat release region range from 250 °C to 430 °C, in agreement with the TG analysis [40]. At the temperature range from 250 °C to 430 °C, the HRR of TLPCF had a sharp increase from lower than 10 W/g to about 173 W/g at 358.8 °C, showing the decomposition of cotton cellulose chains and burning of the pyrolytic micromolecules. As shown in Table 2, the decomposition temperature of the control cotton fabric reached a maximum at 369.6 °C with the HRR at 300.2 W/g (PHRR). Significant changes in the microscale combustion parameters appeared when the cotton fabric was modified with TLP; the temperature was decreased to 358.8 °C, the PHRR and THR decreased to 173.2 W/g (42.3% reduction) and 11.3 KJ/g (13.7% reduction). Thus, the MCC data show that the TLP had a significant impact on the flammability of cotton fabric by increasing cellulose dehydration and promoting char formation [41].

### 3.5. Mechanical Properties

As shown in Table 3, compared with control cotton fabric, the tensile strength and elongation at break of modified cotton fabrics in warp direction decreased slightly, ascribing to the high temperature and long-term treatment of the modification process, but the mild decrease did not affect its normal use. The air permeability of the modified cotton fabrics also had a slight decrease due to the reduction of interweaved interspaces between cotton fibers derived from the chloropyrimidine films formed on the surface of fibers. It was interesting that the whiteness of TLPCF and DMPCF improved but the whiteness of ADPCF decreased sharply because the yellow color of the ADP reagent itself affected the whiteness.

The bending properties and surface friction properties are also important mechanical features that determine fabric drapability and handle [42]. Sensory tests conducted by KES-F instrumental measurements were performed in order to determine the fabric handle, which is a critical physical property for people to make objective decisions [13,43]. The test results of these properties are presented in Table 4.

Bending properties, including bending rigidity (B) and bending moment (2HB), depend on bending resistance and friction between fibers and yarns, as well as fabric structure. B is the ability of a fabric to resist the bending moment. 2HB describes the bending behavior of a fabric subjected to an external load applied perpendicular at the curvatures from 2.5 cm^−1^ to −2.5 cm^−1^ to a longitudinal axis of the fabric [44,45]. Generally speaking, fabrics with low B and 2HB values have better bending properties. Table 4 shows that TLPCF, DMPCF, and ADPCF have an increase in B and 2HB values compared with the control fabric, which can be primarily attributed to the enhancement of interyarn friction and the number of fibers contacting at yarn crossover points in the modified cotton fabrics [13]. In addition, different changes are related to the type of chloropyrimidine compound applied. The B and 2HB values of the modified cotton fabrics were in the following order: ADPCF > DMPCF > TLPCF. Furthermore, an alkaline reaction medium could result in brittleness, making the modified cotton fabrics stiffer. However, the bending properties of TLPCF and DMPCF had no obvious change and maintained a soft and smooth handle. 

Surface friction properties include the friction coefficient (MIU) and the geometrical roughness (SMD). Fabrics with low MIU and SMD values have better surface properties. As shown in Table 4, the MIU and SMD values of TLP modified cotton fabrics decreased a little, which means the surface of TLP modified cotton fabric is smoother and finer than control cotton fabric. The surface roughness reduction was mainly attributed to the films formed on the surface of modified fibers. However, in comparison with control cotton fabric, the increase in the MIU values of DMPCF and ADPCF was due to the residue of macromolecules on the fabric surface. In a word, the roughness of cellulose fibers after modification with chloropyrimidine compounds underwent no significant change.

### 3.6. Dyeing Properties

The cotton fabrics were dyed with Reactive Red 3BF, Yellow 3RF, and Blue KN-R, respectively. Table 5 presents the K/S values, color parameters, rubbing fastness, and washing fastness of control and modified cotton fabrics. It can be seen that the K/S values of modified cotton fabrics dyed with three kinds of reactive dyes increased significantly, indicating that the modification could increase the functional groups that could react with reactive dyes in cotton fabrics. All of the modified cotton fabrics dyed with Reactive Red 3BF showed similar L*, a*, b*, C*, and H values, which meant the modification had little effect on the dyeing of cotton fabrics. For Reactive Yellow 3RF, the color parameters of DMPCF and ADPCF were higher than that of control cotton fabrics, showing greater brightness and chroma. For Reactive Blue KN-R, the L* of all modified cotton fabrics was lower than that of control cotton fabric, indicating higher brightness of modified cotton fabrics. As shown in Table 5, the washing fastness (fastness and staining) of dyed TLPCF, DMPCF, and ADPCF was at the same level as that of control cotton fabric. Because the washing fastness level of the control cotton was already at an excellent level, it could be maintained by the modification process using chloropyrimidine compounds emulsions [46]. In addition, the modified cotton fabrics dyed with Reactive Red 3BF showed a better wet rubbing fastness because of the covalent bonding between C–Cl of chloropyrimidine compounds and –OH of cotton fabric, which improved the adsorption capacity and binding force of pyrimidine compounds on cotton fibers. The modified cotton fabrics could interact with reactive dye to form complex compounds. Consequently, the graft modification of chloropyrimidine compounds did not affect the dyeing fastness of cotton fabrics and even improved the wet rubbing fastness.

## 4. Conclusions

In this study, cotton fabrics were modified with chloropyrimidine compounds (TLP, DMP, and ADP) to improve the antipilling performance. The optimized factors of the modification process were successfully obtained as follows: concentration of chloropyrimidine compounds was 3.0 g/L, concentration of Na_2_CO_3_ and Na_2_SO_4_ were 3.0 g/L, modification temperature was 95 °C, and modification time was 45 min. The results suggested that the modification process caused no significant damage to the physical and mechanical properties of cotton fabrics. After modification with TLP and DMP emulsion systems, the cotton fabrics achieved excellent and durable pilling resistance and improved the pilling resistance by two or more grades before and after washing. Meanwhile, the modified cotton fabrics exhibited good handle, whiteness, heat release behavior, and dyeing properties. This heterogeneous modification strategy could also be applied to other cellulosic and protein materials for further development of multifunctional textiles in the future.

## Figures and Tables

**Table 1:** Pilling grade of cotton fabric before and after five and ten washing cycles.

Pilling Grade	a	b	c	d
Before washing	2–3	5	4–5	5
5 times washing	2	4	3–4	4
10 times washing	1–2	3–4	3	3–4

**Table 2:** Microscale combustion properties of control and modified cotton fabrics.

Sample	△m (%)	HRC (J/ (g. K))	PHRR (W/g)	THR (KJ/g)	T (°C)
CF	87.0	300	300.2	13.1	369.6
TLPCF	85.8	173	173.2	11.3	358.8
DMPCF	74.5	216	215.4	12.1	368.0
ADPCF	75.2	228	228.0	12.4	364.4

**Table 3:** Tensile strength, elongation at break, whiteness, and permeability of control and modified cotton fabrics.

Sample	Tensile Strength (N)	Elongation at Break (%)	Whiteness (%)	Permeability (mm/s)
CF	590.13	10.24	78.6	235.8
TLPCF	542.33	9.65	81.2	228.9
DMPCF	552.36	10.65	78.4	187.8
ADPCF	535.56	9.17	30.2	190.0

**Table 4:** Bending properties and surface friction properties of control and modified cotton fabrics.

Sample	B (gf cm^2^/cm)		2HB (gf cm/cm)		MIU		SMD (μm)	
	Warp	Weft	Warp	Weft	Warp	Weft	Warp	Weft
CF	0.0627	0.0348	0.0741	0.0405	0.159	0.157	4.508	2.990
TLPCF	0.0638	0.0352	0.0819	0.0423	0.152	0.159	4.370	2.540
DMPCF	0.0697	0.0377	0.0824	0.0433	0.179	0.180	3.527	3.598
ADPCF	0.0731	0.0354	0.1040	0.0488	0.183	0.159	3.997	3.513

**Table 5:** K/S value, color parameters, and colorfastness of dyed control and modified cotton fabrics.

Dyestuff	Sample	K/S	Color Parameter					Rubbing Fastness		Washing Fastness		
			L*	a*	b*	C*	H	Dry	Wet	Staining (Cotton)	Staining (Wool)	Fastness
Reactive red 3BF	CF	6.37	50.69	57.04	−4.87	57.24	355.13	4	3	4–5	4–5	4
	TLPCF	6.42	50.87	57.18	−4.76	57.38	355.24	4	3–4	4–5	4–5	4
	DMPCF	6.94	50.08	57.65	−4.51	57.83	355.52	4	3–4	4–5	4–5	4
	ADPCF	6.50	50.58	56.82	−4.53	57.00	355.44	4	3–4	4–5	4–5	4
Reactive yellow 3RF	CF	5.73	74.29	21.43	66.07	69.46	72.03	4	3–4	4–5	4–5	4
	TLPCF	5.80	74.06	21.27	65.87	69.22	72.11	4–5	3–4	4–5	4–5	4
	DMPCF	5.76	76.49	25.56	69.35	73.91	69.77	4	3–4	4–5	4–5	4
	ADPCF	5.92	75.73	25.90	69.21	73.90	69.48	4	3–4	4–5	4–5	4
Reactive blue KN-R	CF	4.60	48.38	−2.31	−37.38	37.45	266.46	4–5	3–4	4–5	4–5	4
	TLPCF	5.97	45.17	−1.03	−39.16	39.17	268.50	4–5	3–4	4–5	4–5	4
	DMPCF	5.76	45.95	−1.82	−37.81	37.86	267.25	4–5	3–4	4–5	4–5	4
	ADPCF	5.30	47.17	−2.15	−37.68	37.74	266.74	4–5	3–4	4–5	4–5	4
